# Tumor treating fields in the management of Glioblastoma: opportunities for advanced imaging

**DOI:** 10.1186/s40644-019-0259-8

**Published:** 2019-11-29

**Authors:** Vikram S. Soni, Ted K. Yanagihara

**Affiliations:** 1 New York Presbyterian – Brooklyn Methodist Hospital, 506 Sixth St., Brooklyn, NY 11215 USA; 2 University of North Carolina, 516 S. Van Buren Rd, Eden, N.C. 27288 USA

**Keywords:** Glioblastoma, Tumor treating fields, Biomarkers, Radiomics

## Abstract

Alternating electric fields have been successfully applied to cancer cells in-vitro to disrupt malignant progression and this antimitotic therapy has now been proven to be efficacious in Phase II and Phase III randomized clinical trials of patients with glioblastoma. With additional clinical trials ongoing in a number of other malignancies, there is a crucial need for a better understanding of the radiographic predictors of response and standardization of surveillance imaging interpretation. However, many radiologists have yet to become familiarized with this emerging cancer therapy and there is little active investigation to develop prognostic or predictive imaging biomarkers. This article provides an overview of the pre-clinical data that elucidate the biologic mechanisms of alternating electric fields as a cancer therapy. Results from clinical trials in patients with glioblastoma are then reviewed while elaborating on the several limitations to adoption of this promising line of treatment. Finally, a proposal for the development of imaging markers as a means of overcoming some of these limitations is made, which may improve treatment utilization by augmenting patient selection not only in glioblastoma, but also other malignant conditions for which this therapy is currently being evaluated.

## Background

Glioblastoma (GBM) is the most common primary malignant brain tumor and despite having a historically dismal prognosis, some gains have been made [1–3]. Temozolomide (TMZ) chemotherapy and the application of externally applied antimitotic tumor-treating fields (TTFields) represent the most notable clinical advances resulting from randomized clinical trials over the past twenty years. However, while TMZ is utilized in essentially all GBM patients fit for treatment, TTFields have been much less widely adopted despite both interventions having the support of national guidelines [4]. One explanation for this limited implementation is a poor understanding of the imaging features associated with TTField response and the absence of any predictive neuroimaging markers. At present, patients are advised to wear this somewhat unorthodox and cumbersome treatment device for some predetermined period or until there is clinical progression of disease, but without any way of predicting or monitoring efficacy. Despite the need for better imaging assessment of TTField therapy, many academic radiologists may be unaware of this therapeutic advance or how prognostic and predictive radiographic biomarkers may aid clinical decisions. In this manuscript, we seek to familiarize radiologists with TTFields by reviewing the pre-clinical data supporting this as an anti-cancer therapy and the clinical studies demonstrating their efficacy in patients with GBM. We also elaborate on several challenges to these data and provide some insights into the reluctance for general adoption of TTFields in clinical practice. Finally, we propose that advances in neuroimaging being applied in many other areas of brain cancer as prognostic and predictive markers have an opportunity to aid in patient selection and follow-up for those undergoing treatment with TTFields. While this review focuses on neuroimaging in patients with GBM, as this is the only FDA-approved application of TTFields, numerous trials evaluating this novel form of therapy are in various stages of accrual for a variety of other disease sites (Table 1) and improved imaging biomarkers will be needed outside the central nervous system.

**Table 1:** Registered trials for all diagnoses that are planned, accruing, or closed to accrual and incorporate TTFeilds

	Title	Phase	Status	NCT Number
*CNS*				
Tumor Treating Fields With Chemoradiation in Newly Diagnosed GBM		I	Not yet recruiting	NCT03705351
NovoTTF-200A and Temozolomide Chemoradiation for Newly Diagnosed Glioblastoma		I	Recruiting	NCT03232424
Safety and Immunogenicity of Personalized Genomic Vaccine and Tumor Treating Fields (TTFields) to Treat Glioblastoma		I	Recruiting	NCT03223103
Enhancing Optune Therapy With Targeted Craniectomy		I	Recruiting	NCT02893137
Study of Marizomib With Temozolomide and Radiotherapy in Patients With Newly Diagnosed Brain Cancer		I	Active, not recruiting	NCT02903069
Temozolomide, Radiation Therapy, and Tumor Treating Fields Therapy in Treating Participants With Glioblastoma		I	Recruiting	NCT03477110
TTFields and Pulsed Bevacizumab for Recurrent Glioblastoma		II	Recruiting	NCT02663271
Study Testing The Safety and Efficacy of Adjuvant Temozolomide Plus TTFields (Optune) Plus Pembrolizumab in Patients With Newly Diagnosed Glioblastoma (2-THE-TOP)		II	Recruiting	NCT03405792
Optune Plus Bevacizumab in Bevacizumab-Refractory Recurrent Glioblastoma		II	Active, not recruiting	NCT02743078
A Phase II Study of NovoTTF-200A Alone and With Temozolomide in Patients With Low-Grade Gliomas		II	Recruiting	NCT02507232
Optune Delivered Electric Field Therapy and Bevacizumab in Treating Patients With Recurrent or Progressive Grade 2 or 3 Meningioma		II	Recruiting	NCT02847559
NovoTTF-100A With Bevacizumab (Avastin) in Patients With Recurrent Glioblastoma		II	Recruiting	NCT01894061
Trial of Combination TTF (Optune), Nivolumab Plus/Minus Ipilimumab for Bevacizumab-Naive, Recurrent Glioblastoma		II	Recruiting	NCT03430791
A Phase II Study of Optune (NovoTTF) in Combination With Bevacizumab (BEV) and Temozolomide (TMZ) in Patients With Newly Diagnosed Unresectable Glioblastoma (GBM)		II	Recruiting	NCT02343549
Effect of NovoTTF-100A Together With Temozolomide in Newly Diagnosed Glioblastoma Multiforme (GBM)		III	Completed	NCT00916409
Effect of NovoTTF-100A in Recurrent Glioblastoma Multiforme (GBM)		III	Completed	NCT00379470
Assessment of Optune Therapy for Patients With Newly Diagnosed Glioblastoma Using Advanced MRI		IV	Recruiting	NCT03297125
Optune (NOVOTTF-100A), Bevacizumab, & Hypofractionated Stereotactic Irradiation In Bevacizumab-Naive Recurrent Glioblastoma (GCC 1344)		N/A	Recruiting	NCT01925573
NovoTTF Therapy in Treating Patients With Recurrent Glioblastoma Multiforme		N/A	Recruiting	NCT01954576
TTFields Together With Temozolomide and Radiotherapy in Patients With Newly Diagnosed GBM		N/A	Active, not recruiting	NCT03780569
Pilot Study of Optune (NovoTTF-100A) for Recurrent Atypical and Anaplastic Meningioma		N/A	Active, not recruiting	NCT01892397
Use of Optune TTF With Radiation as an Alternative for Elderly Patients With Primary CNS Lymphoma		N/A	Not yet recruiting	NCT03530605
Study Of NOVOTTF-200A In Bevacizumab-Naive Subjects With Recurrent Grade III Malignant Astrocytoma		N/A	Not yet recruiting	NCT03450850
NovoTTF-200A Device in Treating Patients With Newly Diagnosed High Risk Oligodendroglioma		N/A	Recruiting	NCT03353896
Feasibility Trial of Optune for Children With Recurrent or Progressive Supratentorial High-Grade Glioma and Ependymoma		N/A	Recruiting	NCT03033992
*GI*				
Study of the NovoTTF-100 L System to Enhance Antitumor Activity in Patients With Predominant Hepatic Metastatic Cancer		I	Not yet recruiting	NCT03203525
Safety Feasibility and Effect of TTFields (150 kHz) Concomitant With Gemcitabine or Concomitant With Gemcitabine Plus Nab-paclitaxel for Front-line Therapy of Advanced Pancreatic Adenocarcinoma (PANOVA)		I/II	Active, not recruiting	NCT01971281
Effect of Tumor Treating Fields (TTFields, 150 kHz) Concomitant With Sorafenib For Advanced Hepatocellular Carcinoma (HCC) (HEPANOVA)		II	Recruiting	NCT03606590
Effect of Tumor Treating Fields (TTFields, 150 kHz) as Front-Line Treatment of Locally-advanced Pancreatic Adenocarcinoma Concomitant With Gemcitabine and Nab-paclitaxel (PANOVA-3)		III	Recruiting	NCT03377491
*GYN*				
Safety, Feasibility and Effect of TTFields (200 kHz) Concomitant With Weekly Paclitaxel in Recurrent Ovarian Carcinoma (INNOVATE/ENGOT-ov50)		I/II	Recruiting	NCT02244502
Effect of Tumor Treating Fields (TTFields, 200 kHz) Concomitant With Weekly Paclitaxel for the Treatment of Platinum-resistant Ovarian Cancer (PROC) (ENGOT-ov50 / GOG-3029 / INNOVATE-3)		N/A	Recruiting	NCT03940196
*LUNG*				
NovoTTF-100 L in Combination With Pemetrexed (Alimta) for Advanced Non-small Cell Lung Cancer		I/II	Completed	NCT00749346
Safety and Efficacy of TTFields (150 kHz) Concomitant With Pemetrexed and Cisplatin or Carboplatin in Malignant Pleural Mesothelioma (STELLAR)		II	Completed	NCT02397928
Effect of Tumor Treating Fields (TTFields) (150 kHz) Concurrent With Standard of Care Therapies for Treatment of Stage 4 Non-small Cell Lung Cancer (NSCLC) Following Platinum Failure (LUNAR)		III	Recruiting	NCT02973789
*METS*				
Effect of TTFields (150 kHz) in Non-small Cell Lung Cancer (NSCLC) Patients With 1–5 Brain Metastases Following Optimal Standard Local Treatment (COMET)		II	Active, not recruiting	NCT01755624
Effect of TTFields (150 kHz) in Non-small Cell Lung Cancer (NSCLC) Patients With 1–10 Brain Metastases Following Radiosurgery (METIS)		III	Recruiting	NCT02831959
Tumor-Treating Fields Therapy in Preventing Brain Tumors in Participants With Extensive-Stage Small Cell Lung Cancer		N/A	Recruiting	NCT03607682
Radiosurgery Plus NovoTTF-200A for Metastatic Small Cell Lung Cancer to the Brain (RAD 1704)		N/A	Recruiting	NCT03488472
*SKIN*				
Optune Device - TTField Plus Nivolumab and Ipilimumab for Melanoma With Brain Metastasis		II	Not yet recruiting	NCT03903640

Abbreviations: *CNS* Central Nervous System, *GI* Gastrointestinal, *GYN* Gynecologic, *LUNG* Small-cell or Non-Small Cell Lung Cancer, *METS* Brain Metastases

### Pre-clinical data

**Fig. 1:**
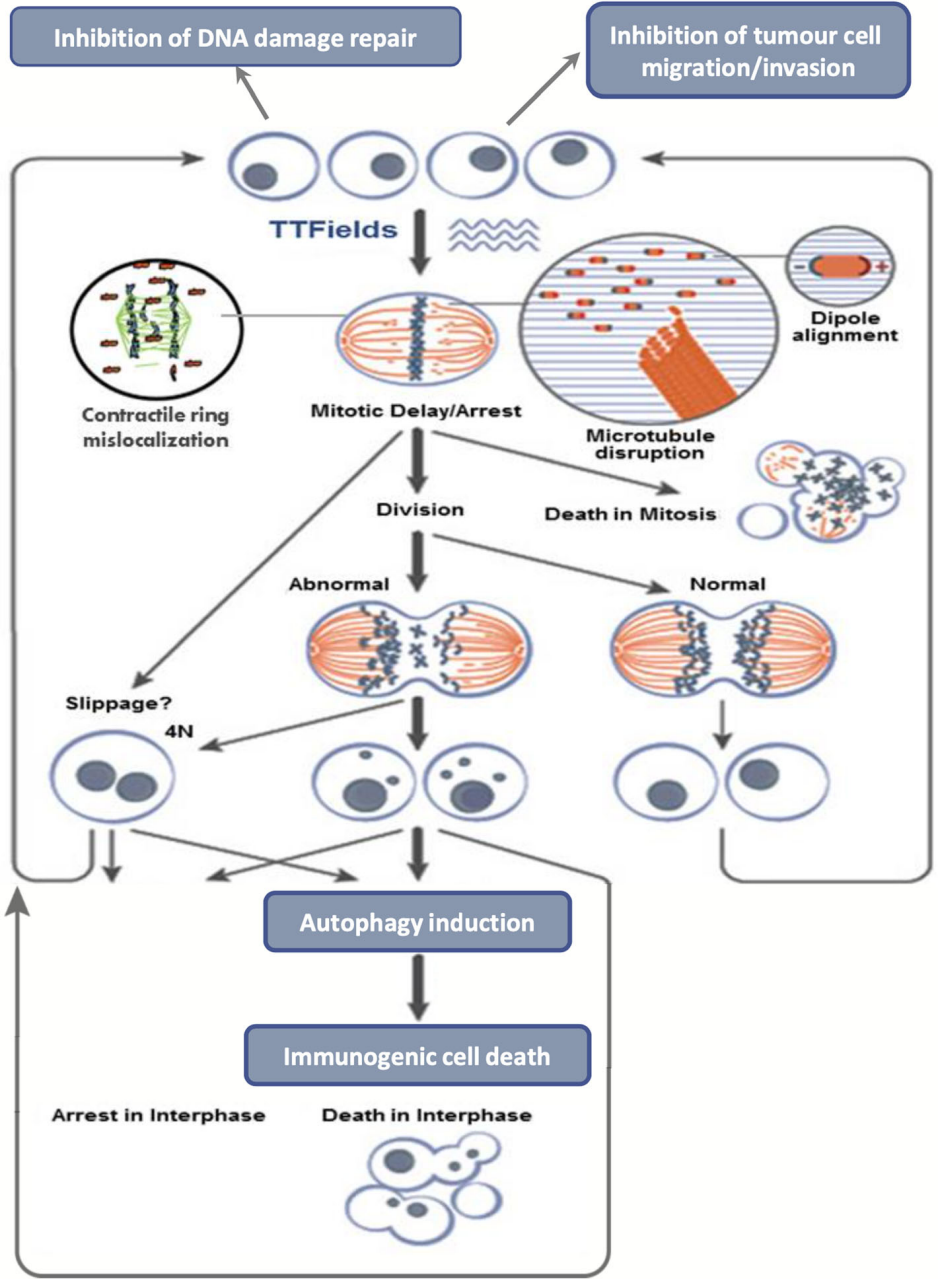
Proposed cellular mechanisms underlying the therapeutic effect of TTFields. In vitro and in vivo experiments have demonstrated that alternating electric fields in the frequency range of 100–300 kHz have specific effects by prolonging mitosis, which leads to the arrest of cell proliferation and may induce cell membrane rupture at the time of cleavage. There appears to be a central reliance on a structural disruption of normal mitotic segregation of cellular components by impairing dipole alignment, which is normally integral in the orderly process of chromosome alignment and separation

Alternating electric fields (AEFs), when applied to living tissue, are known to have a wide range of biological effects [5, 6] (Fig. 1). Applying low-frequency AEFs, on the order of < 1 kHz, to living tissue can stimulate excitable cells through membrane depolarization and it has been theorized that such effects may be harnessed for various medical applications – such as stimulating bone growth and accelerating fracture healing. As the frequency of AEFs increases above 1 kHz -- towards 10 kHz and beyond -- the stimulatory effects on cells appears to diminish, owing to cell membrane hyperpolarization and resistance to further excitation. On this end of the spectrum, at very high frequencies, a different biologic effect is observed – that of tissue heating due to dielectric losses. This phenomenon of tissue heating in response to very high frequency AEFs serves as the basis of several medical treatment modalities, most notably radiofrequency tumor ablation [7].

Although cell excitation and tissue heating have been observed at very low and very high frequency AEFs, respectively, it had previously been thought that intermediate-frequency AEFs, between 100 kHz to 1 MHz, exerted no meaningful biological effect. However, subsequent experiments have in fact refuted this theory, as microscopic particle alignment and cellular rotation have been observed as a consequence of application of low- to intermediate-frequency AEFs [7].

It was on this basis that Kirson et al. [7] experimented with the application of intermediate-frequency, low-intensity AEFs, in the range of 100–300 kHz and < 2 V/cm, on both in vitro and in vivo tissue. The in vitro studies examined the effects of intermediate-frequency AEFs on several cancer cell lines, including malignant melanoma, glioma, lung, prostate, and breast cancer lines. By applying AEFs to these cells, the investigators were able to demonstrate that intermediate-frequency AEFs lead to arrest of cancerous cell proliferation and promoted cell destruction. They also applied these intermediate-frequency AEFs in vivo on two mice tumor models, malignant melanoma and adenocarcinoma. Application of AEFs resulted in significantly smaller tumors compared to control. Given their effect on cancerous cells, these intermediate-frequency AEFs were thus termed tumor-treating fields (TTFields).

What is the underlying cellular basis of these observations? Using time-lapse microphotography during the in vitro experiments, two predominate effects were observed in the TTField-treated cell lines compared to control: prolongation of mitosis leading to proliferation arrest and cell membrane rupture at time of cleavage furrow separation [7]. Further investigation by staining cells with monoclonal antibodies against microtubules, actin filaments, and DNA revealed abnormal mitosis in over half of cell treated with TTFields. This observation may serve as an indication that TTFields interfere with the normal behavior of microtubules – in particular the ordered process of assembly and disassembly of microtubules that is essential for chromosome alignment and separation -- which can explain the mitotic arrest and cell destruction seen in TTField-treated cells [8, 9]. Different cancer cell lines (melanoma, glioma, adenocarcinoma, etc.) demonstrated differing degrees of proliferation arrest and destruction in response to the application of TTFields, but all lines demonstrated statistically significant growth inhibition compared to control [7].

The initial 2004 study was followed by a subsequent study in 2007 by the same study group [10]. The follow up study included additional human cancer cell lines, animal models, and also extended the investigation to include a small pilot trial of 10 patients with recurrent GBM. In this follow up study, a dose- and frequency-dependent response to TTFields in cancerous cells was described (i.e. optimal TTField intensity and frequency for maximal proliferation arrest and cell destruction varied from cell line to cell line). In general, optimal frequencies ranged between 100 and 200 kHz and intensities around 2 V/cm. The investigators were also able to show that the inhibitory effect of TTFields could be improved based on the orientation of the fields and that increasing the number of field directions also significantly increased the anti-proliferative efficacy of the intervention. As mentioned, this study also incorporated a small pilot study of 10 patients with recurrent GBM who were treated with TTFields. Median time to disease progression (TTP) and overall survival (OS) were 26.1 weeks and 62.2 weeks, respectively, more than double reported historical controls [11–15]. In terms of patient safety, there were no serious adverse events and no significant changes in serum chemistry or blood counts. Nine out of the 10 patients developed mild contact dermatitis at the site of electrode placement, which responded well to the application of steroid creams and repositioning of electrodes.

A subsequent pilot study was conducted in 2008 [16] in which six patients with varying solid tumor malignancies who had exhausted all standard treatment lines were treated with application of TTFields via insulated external electrodes. Two patients were treated for two weeks continuously, four patients were treated for four weeks continuously. Patients were allowed to disconnect from the device up to 30 min twice a day. This study demonstrated that TTFields were generally well tolerated, with the only adverse effect again being grade 1 dermatitis of the scalp.

In 2009 Kirson et al. [17], published another series of experiments, in vivo, in vitro, and in 20 patients with GBM as a pilot study, to investigate the safety and efficacy of combining TTFields with chemotherapy. In both the in vivo and in vitro studies, the combination of TTFields with chemotherapy significantly reduced cancer cell proliferation compared against control and either TTFields or chemotherapy used alone. In the pilot study, 20 GBM patients were included and treated with TTFields continuously for an average of one year. Ten patients with disease recurrence after receiving standard upfront therapy were treated with TTFields alone and 10 newly diagnosed patients who underwent surgical resection and adjuvant chemoradiation with TMZ were treated with a combination of TTFields and maintenance TMZ. In the recurrent GBM group treated with TTFields alone as salvage therapy, both progression-free survival (PFS) and OS were significantly higher than historical controls and similar to prior data. In the newly diagnosed GBM group, the combination of TTFields and maintenance TMZ after surgery and chemoradiation demonstrated a median PFS of 155 weeks and a median OS of > 39 months, both considerably improved compared to historically reported data of patients receiving maintenance TMZ alone. Of note, the improvement in PFS and OS with the addition of TTFields to maintenance TMZ did not result in any increased treatment toxicity. Again, the primary device-related toxicity was scalp dermatitis which was managed with topical steroid treatment and electrode repositioning and completely resolved within days to weeks of treatment completion.

### Clinical data

The promising results of the above pre-clinical data and pilot trials lead to the first randomized phase III study of TTFields in recurrent GBM, the EF-11 trial [18]. In this trial, 237 patients with radiologically confirmed disease progression (by Macdonald criteria [19]) who had at least received prior radiation treatment were randomized to receive either TTField monotherapy or physicians choice best active chemotherapy. There were 120 patients allocated to the TTField arm and 117 to the chemotherapy arm. Of note, in both groups, roughly 45% of the patients were being treated for their second recurrence and for approximately 40% of patients it was their third. Mean usage of the TTFields device in the experimental arm was 20.6 h per day. At median follow up of 39 months, 220 patients (93%) had died. Median survival was marginally higher in the TTField arm compared to chemotherapy at 6.6 versus 6.0 months, respectively. Median PFS was also comparable between the two groups at 2.2 months in the TTField group and 2.1 months for the chemotherapy arm [18]. Hazard ratio for death was 0.86 (CI 0.66–1.12) in favor of the TTField arm and more objective radiographic responses (i.e. complete and partial responses) were seen in the TTField group compared to the chemotherapy group: 14 versus 7, respectively. In terms of safety, 16% of patients treated with TTField experienced mild to moderate scalp dermatitis, the expected side effect of TTField. Patients receiving active control chemotherapy experienced side effects related to the chemotherapy agent used. Quality of life (QOL) was analyzed for patients who remained on study therapy for > = 3 months and for whom QOL data was available (63 patients, 27%). No meaningful differences were seen between TTField and active chemotherapy in the domains of global health and social functioning. Cognitive and emotional functioning, as well as role functioning favored TTField, whereas physically functioning slightly favored chemotherapy [18]. Although a negative trial, the results of the EF-11 trial demonstrated that TTField therapy was safe and efficacious compared to chemotherapy and lead to the approval in the US and Europe of TTFields in the setting of recurrent GBM.

These results, as well as pilot data demonstrating feasibility of combining TTFields with TMZ [17], ultimately lead to the EF-14 trial -- a phase III randomized trial evaluating TTFields in patients with newly-diagnosed GBM after completion of chemoradiation [20]. In this trial patients were eligible who had histologically confirmed supratentorial GBM and who were progression-free after maximal safe resection or biopsy and completion of standard concomitant chemoradiation with TMZ. Patients were then randomized to receive standard maintenance chemotherapy with TMZ with or without TTFields. Here, the placement of transducer arrays was optimized to cover the target area using mapping software (NovoTAL, Novocure Inc) based on each individual’s post-surgery MRI. A preplanned interim analysis of the first 315 patients enrolled found both a significant PFS and OS benefit favoring the group who received TTFields with TMZ, compared to TMZ alone. The benefit was considerable enough to prompt the independent data and safety monitoring committee to recommend early termination of the trial and ultimately led the FDA to expand the approval of TTFields in the setting of newly diagnosed GBM.

The final analysis involving all 695 randomized patients from EF-14, with median follow up of 40 months, was reported in 2017 [1]. The post-hoc analysis revealed that TTFields plus TMZ was associated with an increase in PFS and OS in all patient subgroups -- regardless of age, sex, MGMT promoter methylation status, Karnofsky performance status, extent of resection or geographic location. The addition of TTFields to TMZ, compared to TMZ alone, resulted in longer median PFS from the time of randomization -- 6.7 verse 4.0 months, respectively -- as well as prolonged OS, 20.9 verse 16.0, respectively. The rates of systemic adverse effects were not significantly different between the two treatment arms, however there was a higher incidence of mild to moderate scalp irritation in the group receiving both TTFields plus TMZ.

### Quality of life under treatment with TTFields

Despite advances in treatment over the years, the prognosis for GBM patients remains unfortunately poor, with median OS of about 14 months in the clinical trial setting and 11 months in the general GBM population [2, 21, 22]. Furthermore, even after multimodal therapy patients experience recurrence within seven months on average [1]. Given this grim outlook, novel treatment approaches and clinical trials have not only focused on improvements in progression-free and OS but have also highlighted the effects on symptom palliation and improving patient functioning. As such, assessment of neurocognitive functioning and health-related quality of life has become a priority in GBM-trials.

For patients in the EF-11 and EF-14 trials discussed above, TTFields were administered via placement four transducer arrays, each consisting of nine insulated electrodes, directly on the shaved scalp. The transducer arrays were in turn connected to a portable device set to generate 200-kHz electric fields within the brain. Patients were trained on transducer array placement and instructed to replace the transducer arrays twice per week. Uninterrupted treatment was recommended, and patients were expected to use the TTField device continuously to reach a goal of 18 h per day in a four-week period, which is equivalent to 75% of the time. Patients had to carry the electric field generating device, housed in a small pack weighing approximately six pounds, with them while wearing the device (of note, a second-generation device has now come to market that is less than half the weight and size of the original device). Given all of the above, it seems reasonable to question the impact treatment by TTFields would have on patient’s overall quality of life.

In order to assess the effect of TTField treatment on quality of life, patients in the EF-14 trial were assessed by way of measurement of KPS, and by completion of questionnaires assessing cognitive functioning and health related quality of life via the Mini-Mental Status Exam (MMSE) and an EORTC quality-of-life questionnaire core-30 (QLQ-C30, version 3), supplemented by the brain cancer module (BN20). MMSE and KPS assessments were made within 1 week of treatment initiation and then repeated once monthly throughout treatment. Similarly, HRQoL questionnaires were administered at treatment initiation and again every three months until progression or withdrawal from the trial. In 2017 Zhu et al. published the analysis of quality of life outcomes from the interim analysis of EF-14 [23]. This interim review demonstrated no significant differences across any of the outcome measurements -- including KPS-assessed functional status, mean MMSE-measured cognitive status, and HRQoL -- between those patients treated with combination TTFields/TMZ or TMZ alone from baseline to month 12. Symptoms known to be associated with TMZ treatment, including nausea, vomiting, diarrhea, loss of appetite, fatigue, etc., were reported in both groups, no differences in rates or severity were noted between the two groups. Group differences were pronounced for “itchy skin,” which was reported at higher rates for the TTFields/TMZ group. Subsequent analysis of the final trial data found similar results with respect to HRQoL [24]. No difference was found between the groups with respect to HRQoL except for the skin reaction, which was mild to moderate in 52% of TTField /TMZ-treated patients and severe in 2%. Global health status and social functioning were not significantly different between the groups. Role/social functioning and physical functioning were also not significantly different.

### Interpreting the poor adoption of TTFields

Despite the totality of results between the EF-11 and EF-14 trials - the PFS and OS benefits favoring TTFields as well as the lack of difference in QoL measures - adoption of TTField treatment has not been widespread. Reasons for the perceived lack of enthusiasm are multifaceted [25], owing both to factors related to trial design as well as the inherent challenges related to the treatment of GBM itself. In addition to the TTField device having an unorthodox appearance, skepticism or lack of understanding of the mechanism of action of TTFields is also an important contributing factor. The open-label design and lack of a sham-control arm may raise concerns regarding the validity of the trial results and affect acceptance of the treatment. Perhaps more importantly, questions were raised regarding the generalizability of the TTField trial results to the GBM population at large. Only patients who did not progress after radiotherapy were included in the EF-14 randomization. As such, this cohort represents a particularly favorable subset of the general GBM population, as patients who experienced progression after chemoradiation were excluded. It remains a great challenge to identify risk factors for early progression, and no MGMT-style marker is available to guide clinicians as to which patients may particularly benefit from TTField therapy. As articulated previously [25], in an era where oncology has embraced precision medicine and neuro-oncology looks to build on post-MGMT personalized approaches to GBM [26, 27], the one-size-fits-all application of TTFields has been criticized. It is also true that patients are typically anxious to know if a treatment is having its intended effect and oncologists may find it challenging to explain how a patient might benefit from TTFields, for what duration the treatment should be planned, and what criteria will be used to stop the treatment. Informed patients will also be eager to know if their tumor is more or less likely to respond to TTFields, but there are currently no predictive markers to provide such an assessment.

### Neuroimaging as a potential biomarker for TTField response

Various neuroimaging techniques have been investigated to inform prognosis and provide predictive markers of treatment response in GBM patients. These include positron emission tomography and magnetic resonance-based acquisitions, such as diffusion, spectroscopy, measures of blood volume and blood flow kinetics, along with a variety of post-processing tools for quantification and tracking of treatment-related changes in these measures. To date, there are no predictive neuroimaging tools for patients with GBM that are routinely used in clinical practice. However, several lines of investigation have yielded promising results.

Predictors of local recurrence afford the hypothetical benefit of guiding local therapy, whether by surgery, conventional fractionated radiotherapy, radiosurgery, or TTFields. The simplest method of predicting an area of recurrent tumor is inspection of the post-operative bed for residual disease and evidence supports the practice of characterizing the pattern of signal change in this region [28, 29]. Various computational methods have also been explored to evaluate the pre-operative peritumoral region or predict future recurrence based on subtle post-treatment radiographic changes [30–32]. Further, the combination of multiple imaging modalities [33, 34] might improve prediction specificity. These numerous lines of investigation might influence the application of local therapies, but advanced neuroimaging techniques may also have a role in predicting response to treatment and in this capacity could be applied to TTFields.

**Fig. 2:**
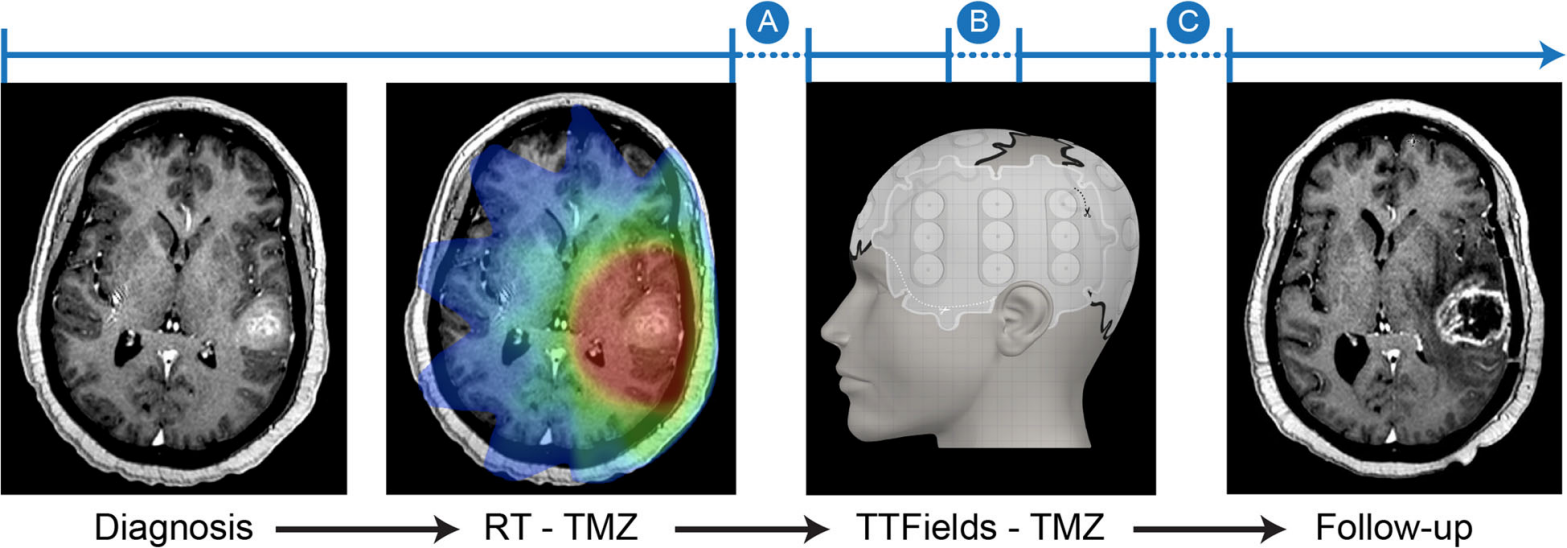
Illustrative treatment time course for a patient with GBM. After a pathologic diagnosis of GBM, patients who are fit for treatment typically undergo a course of combined radiation with concurrent TMZ for a period of three to six weeks. This is followed by a short treatment break of approximately one month followed by TTFields with concurrent TMZ for at least six months or until treatment failure. Breaks in the timeline highlight areas where imaging biomarkers could provide a predictive tool to aid the decision to pursue treatment (Point A), continue treatment via response assessment (Point B), and discontinue treatment due to progression (Point C)

At present, there are no proven neuroimaging tools available to provide response information that is specific to TTFields although there are several points in a patient’s treatment where such information would be highly valuable (Fig. 2). Additionally, mapping studies to determine TTField electrode array placement are based on relatively rudimentary imaging techniques and are determined by a static pre-treatment MRI acquisition without modification according to any radiographic changes while undergoing therapy [35, 36].

As discussed elsewhere, major clinical trials of TTFields have relied on the Macdonald criteria [19] for response assessment [11, 37], while some have argued that the Response Assessment in Neuro-Oncology (RANO) [38] guidelines should be adopted [39]. In perhaps the most detailed review of radiographic changes detected in TTF-responders, Vymazal and Wong [40] made several important observations that provide strong motivation to develop patient-specific predictive markers of TTField efficacy. First, responses seen on serial MRI were highly correlated with OS and compliance to TTFields. This confirms that radiographic improvements in patients undergoing treatment with TTFields are clinically meaningful. However, the authors noted that there was a relatively long interval to response with the median being 5.2 months, which is a considerable length of time to judge treatment efficacy in this patient population. Finally, early pseudoprogression was seen in numerous cases with 44% of responders showing initial tumor growth after starting TTFields. The presence of a long duration before a response can be observed may deter some patients from initiating or continuing therapy and this is compounded by pseudoprogression, where many patients might stop an efficacious therapy on the basis of false-positive imaging results. Therefore, there is an acute need for imaging tools that might predict or detect TTField response with more sensitivity and accuracy.

### Measuring TTField response with amino acid PET

Primary investigations utilizing advanced imaging in patients receiving TTFields have been limited to small series and, to date, most have been reported in abstract form only. Of these, perhaps the most relevant study evaluated the utility of amino acid PET in detecting tumor progression in patients with high-grade glioma [41]. Here, the authors analyzed data from 11 patients with GBM and 1 patient with gliosarcoma whose treatment included the use of TTFields and follow up included *O*-(2-^18^F-fluoroethyl)-L-tyrosine (FET) PET. At the time of their analysis, nine patients were found to have tumor recurrence by either tissue sampling (*n* = 4) or clinically evident progression (*n* = 5). In all nine, FET-PET demonstrated tumor recurrence according to standardized values based on a prior investigation using biopsy-controlled specimens [42]. In addition to demonstrating the feasibility of obtaining quality FET-PET scans in patients treated with TTFields, the authors made a few pertinent observations. Specifically, three patients underwent FET-PET prior to the initiation of TTField therapy and again later when there was tumor progression. In these three, changes in FET-PET parameters (i.e. quantified metabolic activity or tumor/brain ratio) correlated with tumor progression while undergoing TTField therapy. In a separate patient that had not progressed during the study period, FET-PET was obtained prior to initiation of TTFields and then again at 3- and 6-months into therapy. There appeared to be a time-dependent response to treatment in terms of metabolic activity while receiving TTFields. In another patient who showed no signs of clinical progression, comparison of FET-PET scans obtained at baseline and after 5 months of therapy were also objectively unchanged. Finally, contrast-enhanced MRI in one patient suggested tumor recurrence while receiving TTFields, but the response in metabolic activity seen on FET-PET was more consistent with pseudoprogression. With longer follow-up, it was demonstrated that FET-PET had accurately identified pseudoprogression.

In another demonstration of how amino acid PET might be utilized with TTFields, Mittal, et al. [43] recruited patients with recurrent GBM into a prospective study testing the ability of alpha[C-11]-methyl-L-tryptophan (AMT) PET to predict early response to TTField therapy. Five patients who met criteria for analysis had AMT-PET prior to the start of TTFields and then again within 3 months of starting. Patients also underwent at least one contrast-enhanced MRI during that period as part of standard evaluation. The results were encouraging, with four of the five patients showing a metabolic response following initiation of TTFields (with or without concurrent salvage chemotherapy) and the fifth patient experienced progression based on increased metabolic activity and concordant MRI features. The same authors performed a follow-up report with a slightly larger population where a TTField intensity map, simulating a dose distribution, was designed based on a realistic head model created from the patient’s cross-sectional imaging. This three-dimensional electrical field intensity map was then overlaid with the patient’s sequential AMT-PET scans obtained during TTField treatment. In nine patients who met treatment-compliance criteria for analysis, there were 16 tumor regions analyzed. In 13 of these (81%), there appeared to be a metabolic response while undergoing TTField therapy. Furthermore, there was a significant correlation between higher mean electrical field intensities and the magnitude of AMT-PET metabolic response (r = − 0.55, *p* = 0.028). Most of the data supporting amino acid PET is anecdotal and still in abstract form, but the evidence that PET may be sensitive and specific to TTField response and might supplement what is lacking in the interpretation of standard contrast enhanced MRI is thought provoking and warrants further study.

### Measuring TTField response with MRI

A small volume of reports have utilized various MRI-based approaches to quantify changes related to TTFields, but essentially all data are in abstract form and may establish only a proof of principal. The one published comprehensive application of MRI to assess TTField response is described in a case report [44] of a patient with newly diagnosed GBM who was assessed with diffusion, perfusion, and spectroscopic imaging before and again at 1- and 2-months during single-modality treatment with TTFIelds. Multi-parametric MRI results were consistent with a reduction in viable tumor, including an increase in mean diffusivity and a decrease in tumor volume, fractional anisotropy, regional cerebral blood volume, and levels of choline.

Three slightly larger series of patients have also been recently described using diffusion [45, 46], a combination of diffusion and perfusion [47], and one utilizing only volumetric analysis of traditional contrast-enhanced [48] MRI. Noting that one study showed a qualitative, but not statistically meaningful, relationship between radiographic responses to TTFields and clinical outcomes [47], initial results are again encouraging. Specifically, ongoing work by Vymazal and colleagues [45, 46] suggests that changes in the apparent diffusion coefficient and mean diffusivity in patients under treatment with TTFields correlate with OS and PFS. Though preliminary, these findings accord with clinical studies utilizing a similar application and analytic approach to diffusion imaging in GBM patients undergoing treatment with radiotherapy. In such studies, functional diffusion map (fDM) [49, 50] analysis quantifies MR signal change across time points while undergoing treatment either with radiation or chemoradiation. In a prospective trial of patients with newly diagnosed grade III-IV glioma being treated with radiotherapy, fDM analysis performed near the mid-point or after completion of treatment was predictive of OS [51] and this finding was validated in a separate study using a post-treatment timepoint exclusively in GBM patients treated with modern combined chemoradiotherapy [52]. Taken together, these data indicate that certain diffusion parameters may be used as predictive biomarkers in GBM patients undergoing treatment with chemoradiation and preliminary findings suggest that they may also be of clinical value in assessing responses to TTFields.

## Conclusions

For many patients with GBM and their caregivers, surgery, radiation, and chemotherapy are tractable treatments with a proven history in many cancers whereas TTFields are a novel and unorthodox intervention. Despite having a large volume of pre-clinical data, positive results from randomized trials, and national guidelines recommending their use, TTFields have yet to become commonplace. While concerns over QoL have been cited as one reason for limited adoption, recent studies have found no difference in QoL assessments from wearing the device, other than rates related to dermatitis.

Another major limitation relates to the lack of a method to predict or quantify the efficacy of TTFields, which leads to a nearly indefinite and potentially frustrating surveillance period while receiving the treatment. This is perhaps the area of greatest need for future investigation into TTFields in patients with GBM. Throughout oncology, advanced imaging techniques are being studied and applied to provide patient-specific prognostic information and tailor treatment in a dynamic way. For patients with GBM, several imaging tools are proven to be predictive of a response to radiotherapy, but at this time their value in patients undergoing treatment with TTFields is unknown. Systematic review of radiographic information collected as part of prior clinical trials may be informative and prospective acquisition of advanced neuroimaging data in patients being treated with TTFields is strongly encouraged. Whether radiographic markers that have shown promise in patients undergoing treatment with chemoradiotherapy can be successfully applied to TTFields is yet to be determined, but are worthy of future investigation.

## Data Availability

Not applicable.

## Abbreviations

AEFs: Alternating electric fields
CBV: Cerebral Blood Volume
fDM: Functional Diffusion Map
GBM: Glioblastoma
OS: Overall Survival
PFS: Progression Free Survival
QoL: Quality of Life
RANO: Response Assessment in Neuro-Oncology
TMZ: Temozolomide
TTFields: Tumor Treating Fields
